# Personal identification with artificial intelligence under COVID-19 crisis: a scoping review

**DOI:** 10.1186/s13643-021-01879-z

**Published:** 2022-01-06

**Authors:** Shinpei Matsuda, Hitoshi Yoshimura

**Affiliations:** Department of Dentistry and Oral Surgery, Unit of Sensory and Locomotor Medicine, Division of Medicine, Faculty of Medical Sciences, University of Fukui, 23-3 Matsuokashimoaizuki, Eiheiji-cho, Yoshida-gun, 910-1193 Fukui, Japan

**Keywords:** Personal identification, Artificial intelligence, Convolutional neural network, COVID-19 pandemic, Contactless methods, Scoping review

## Abstract

**Background:**

Artificial intelligence is useful for building objective and rapid personal identification systems. It is important to research and develop personal identification methods as social and institutional infrastructure. A critical consideration during the coronavirus disease 2019 pandemic is that there is no contact between the subjects and personal identification systems. The aim of this study was to organize the recent 5-year development of contactless personal identification methods that use artificial intelligence.

**Methods:**

This study used a scoping review approach to map the progression of contactless personal identification systems using artificial intelligence over the past 5 years. An electronic systematic literature search was conducted using the PubMed, Web of Science, Cochrane Library, CINAHL, and IEEE Xplore databases. Studies published between January 2016 and December 2020 were included in the study.

**Results:**

By performing an electronic literature search, 83 articles were extracted. Based on the PRISMA flow diagram, 8 eligible articles were included in this study. These eligible articles were divided based on the analysis targets as follows: (1) face and/or body, (2) eye, and (3) forearm and/or hand. **A**rtificial intelligence, including convolutional neural networks, contributed to the progress of research on contactless personal identification methods**.**

**Conclusions:**

This study clarified that contactless personal identification methods using artificial intelligence have progressed and that they have used information obtained from the face and/or body, eyes, and forearm and/or hand.

**Supplementary Information:**

The online version contains supplementary material available at 10.1186/s13643-021-01879-z.

## Background

Personal identification (PI) indicates techniques and social systems of identification of unidentified persons and identity resolution regardless of whether the persons are alive or dead. PI has been primarily useful in the areas of forensic science and criminal justice [1]. Additionally, PI has become more important in relation to the aging of developed countries and large-scale disasters related to global warming in recent years [1–4]. During the coronavirus disease 2019 (COVID-19) pandemic in 2020, ties between local communities and households have been tested, and households have separated from the safety nets provided by social networks [5]. The importance of PI methods and social systems has increased during this crisis. For timely identification of unidentified persons and bodies, the cooperation of community residents and related experts, such as police officers, rescue teams, medical doctors, and forensic scientists, is critical [1–4]. Further, there is an urgent need to develop objective methods based on digital technologies used for PI and to build a database of the use of these methods as social and institutional infrastructure [6].

Artificial intelligence (AI) is a complex information processing system that has its roots in some aspects of biological information processing [7]. In recent years, AI has made remarkable progress and has been applied in various fields, including the medical field [8]. In the future, not only will it be possible to detect all organic lesions but AI technology will also be helpful in diagnosing psychiatric diseases [9, 10]. In addition, its usefulness in the forensic field has also been reported [11]. AI enables objective PI by analyzing various aspects of personal information of the human body and may contribute to building a personal information database as a component of social infrastructure [12].

COVID-19 is transmitted by respiratory droplets and contact routes [13]. Therefore, during the COVID-19 pandemic, it is an important consideration that there is no contact between the subjects of the PI and the PI systems. In 2014, Sun et al. proposed a remote infection screening system based on multiple vital signs and multivariable logistic regression model and reported that the sensitivity and specificity of this screening method was approximately 80% [14]. Since then, what progress has been made in contactless bioanalytical technologies that could be applied to PI based on AI and could be useful even in the face of widespread infectious diseases such as COVID-19? To the best of our knowledge, the answer to this question has not been investigated in a progress review in the past 5 years.

The aim of this scoping review was to organize the progression of contactless PI methods using AI over the past 5 years and discuss methods for their use during the COVID-19 crisis.

## Methods

The authors conducted a scoping review, which is used to clarify working definitions and conceptual boundaries of a topic or field, following the Preferred Reporting Items for Systematic Reviews and Meta-Analyses extension for scoping reviews (PRISMA-ScR) and adopting The Joanna Briggs Institute methodology framework [15–19] (Additional file 1). The authors have registered the protocol in the Open Science Framework (https://osf.io/wns3e/).

### *Eligibility*, *inclusion*, *and* exclusion criteria

Eligibility criteria were established using the Participant-Concept-Context (PCC) model outlined by The Joanna Briggs Institute guidelines as follows [15–19]: **P**: unidentified persons and bodies; **C**: the development status of AI technology applied for PI under infectious disease pandemic; that is, only the technologies that did not require contact between the unidentified persons and bodies and the analytical instrument were extracted; and **C**: contactless PI methods using AI technology applicable under infectious disease pandemics.

Contactless vital sign monitoring system was reported in 2014 [14]. The recent development of digital technology has been remarkable, and the COVID-19 pandemic has reaffirmed the importance of contactless PI methods. Therefore, only studies published between January 2016 and December 2020 were included in this review to identify recent advances in the field of AI.

The inclusion criteria were as follows: (1) studies on contactless PI methods using AI.

The exclusion criteria were as follows: (1) case reports, case series, reviews, conference papers or proceedings, and letters to the editor; (2) animal experiment trials; (3) unavailability of full text; and (4) articles in languages other than English.

### Information sources and search strategy

An electronic systematic literature search was conducted using the PubMed, Web of Science, Cochrane Library, CINAHL, and IEEE Xplore databases. The literature search strategy is presented in Table 1. The electronic searches were performed on December 31, 2020.

**Table 1:** Electronic literature search strategy

Database	Search strategy	Number of articles
PubMed	((Personal identification [MeSH Terms]) AND (artificial intelligence [MeSH Terms])) AND ((“2016/01”[Date - Publication]: “2020/12”[Date - Publication]))	12
Web of Science	Topic: (personal identification) OR Topic: (artificial intelligence), Document type (Article), Timespan 2016-2020	30
Cochrane Library	Personal identification in Keyword AND artificial intelligence in Keyword	0
CINAHL	Personal identification AND artificial intelligence AND Publishdate(20160101-20201231)	3
IEEE Xplore	Personal identification in All Metadata AND artificial intelligence in All Metadata AND (Filters applied: Journals AND (Publication year: 2016-2020))	38

### Study selection

S.M. and H.Y. independently performed the literature evaluation and selection. Based on the PRISMA flow diagram for the scoping review process, the study selection process consisted of the removal of duplicates, screening of titles and abstracts, and reviewing of full texts [15–19]. Disagreements between reviewers were resolved through discussion and consensus.

### Critical appraisal

The reviewers performed critical quality appraisal of included articles using the mixed method appraisal tool version 2018 [20].

### Data charting process and synthesis of results

According to the guidance for scoping review of Joanna Briggs Institute [15–19], both reviewers (S.M. and H.Y.) independently extracted the following data: author(s), year of publication, PI methods using AI, and the biometric information targeted by those methods. After the data extraction, both reviewers (S.M. and H.Y.) independently categorized and synthesized the data. At each step, disagreements between reviewers were resolved through discussion and consensus.

## Results

### Study selection

**Fig. 1.**
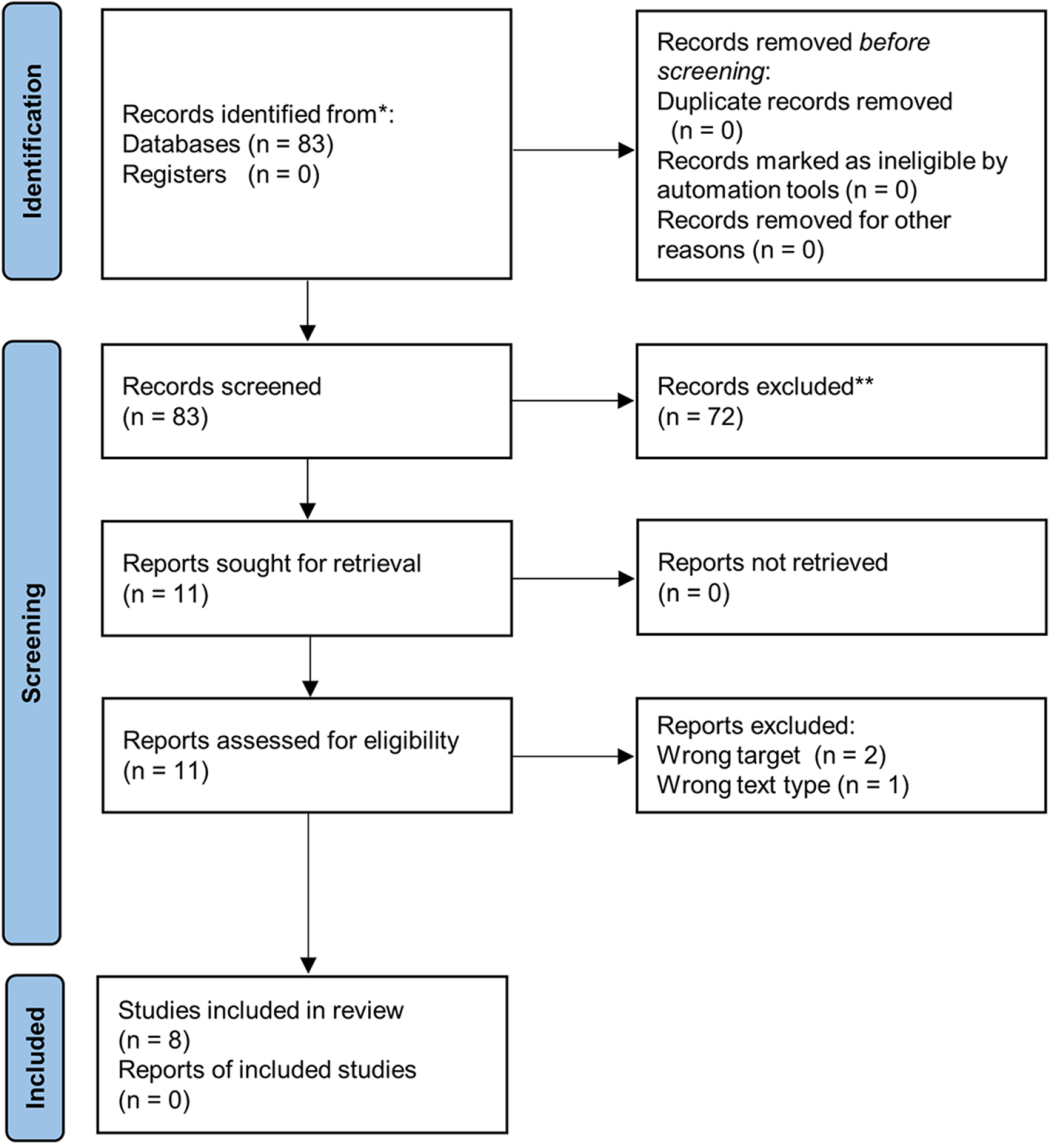
Flow diagram based on the PRISMA flowchart template of the search and selection process

Based on the PRISMA flow diagram, the process of search and selection of studies was performed (Fig. 1). An electronic literature search was performed using the PubMed, Web of Science, Cochrane Library, CINAHL, and IEEE Xplore databases, and 83 articles were extracted. There were no duplicate articles. Based on the PCC eligibility criteria described above, 72 articles were excluded on the basis of their title and abstract. Eleven full articles [21–31] were assessed for eligibility, and the critical appraisal step was performed with each. Finally, 8 eligible articles [21, 22, 24, 26–28, 30, 31] were included in this study. These eligible articles were divided based on the analysis target as follows: (1) face and/or body [21, 22, 26, 27], (2) eye [24, 28], and (3) forearm and/or hand [30, 31]. The details of these studies are described below.

### Face and/or body

The facial region is the most individually recognizable region and has special characteristics and landmarks. The progress of cameras that can capture facial and bodily features accurately has contributed to the development of PI methods [21, 22, 26, 27]. Nguyen and Park reported a gender recognition method using a support vector machine and a local binary pattern [21**].** Much conventional supervised machine learning requires a “gold standard” dataset annotated by human experts [22]. Therefore, Huang et al. reported a user-dependent approach for identifying affective states from spontaneous facial expressions based on multiple-instance learning without the need for expert annotation [22]. In 2017, Ranjan et al. reported an algorithm based on a deep convolutional neural network (CNN) for face detection, landmark localization, pose estimation, and gender recognition [26]. Furthermore, Sang et al. reported a person identification method based on a multi-information flow CNN model and attribute recognition for improving the accuracy of the technology [27]. CNN-based methods have shown high accuracy and have become an indispensable technology in this field.

### Eye

The eye and facial area containing the eye have been used in PI [24, 28]. PI based on iris recognition has been applied in border security control, banking, law enforcement, public welfare distribution, and accounting because each individual has a unique iris pattern [28]. Improvements in accuracy are constantly required since these data are treated as important information for PI. Wang and Kumar proposed an iris recognition method based on a deep CNN with dilated and residual learning [28]. In 2018, Chen et al. reported an end-to-end network based on conditional generative adversarial networks designed to generate face information based only on the eye region and suggested that the method could provide potential for PI even in cases of face occlusion [24].

### Forearm and/or hand

Methods of PI using hand information, including finger/palmprints and blood vessels, are widely accepted in law enforcement and security [30]. However, most of these methods require contact between the body parts and the devices to obtain the information for PI. This may not be applicable in the face of widespread infectious diseases, including COVID-19. Liu and Kumar reported an identification method designed using a contactless palm detector and trained CNN that may have the potential for online PI [30]. Nabulsi et al. reported a noncontact forearm measurement method using microwaves [31]. They suggested that the noncontact microwave biometric information scan technique was useful because of its robustness in the presence of environmental lighting and its unobtrusiveness [31].

## Discussion

This scoping review clarified the recent 5-year progression of contactless PI systems using AI. The analysis targets were divided into three body regions: face and/or body, eye, and forearm and/or hand. These results show that there is no significant change in analysis targets from conventional PI methods. Moreover, this review clarified that PI methods using AI, including CNN architectures, are enabling progress in this area. Based on the collected evidence, scientists continue to recognize the importance of contactless PI during the COVID-19 pandemic and will continue to do so after the pandemic is over. Thus, the progress and commercialization of contactless PI methods will be accelerated. Furthermore, these technologies might build social and institutional infrastructure.

Advances in this digital area constructed with body and facial image acquisition devices and AI have the potential to replace fingerprint-based methods, which have long been used for PI. However, most PI systems based on AI related to information from faces and/or body parts must be trained with images of humans, and selection of images requires involvement of human trainers [29]. Therefore, at this time, it is difficult to completely eliminate planners’ and trainers’ sociocultural biases in facial recognition systems with AI [29]. Moreover, the problem of overfitting is inevitable with the small-scale databases used in these systems [25]. Therefore, the scale of the situation and conditions in which the technology will be applied should be carefully considered.

Recently, research and development of wireless and contactless methods of PI have been actively pursued [21–31]. However, as a limitation of contactless PI methods using cameras to capture body part information, Wu et al. mentioned their sensitivity to illumination changes and visual occlusion [32]. Additionally, accuracy and security need to be considered in signal processing of biometric personal information and in information management, including the steps required to send and receive the information. Therefore, researchers and politicians involved in the development of contactless PI systems for social infrastructure must work to improve the accuracy of PI methods and determine how to handle biometric information, register it in databases, and use it. It is also necessary to reach a consensus on how to obtain informed consent from individuals (from parents or caregivers in case of minors) to be registered in the database and the use of their information.

The World Health Organization (WHO) described *COVID-19 as the disease caused by a new coronavirus called SARS-CoV-2. WHO first learned of this new virus on December 31, 2019* [13, 33]. Therefore, man is not fully equipped with COVID-19 countermeasures, though responses are being updated daily. The global spread of infectious diseases could hinder the achievement of the 17 Sustainable Development Goals announced by the United Nations in 2015 in the targets of health, poverty, communities, and others [34]. Medical experts need to make efforts to collect appropriate information on infectious diseases, including COVID-19, and scientists need to make efforts to develop PI methods based on such information. The contactless system for the collection of PI information will be able to play a central role in this process and in the achievement of the Sustainable Development Goals.

## Conclusions

This scoping review clarified the recent 5-year progression of contactless PI systems using AI, which have used information from the face and/or body, eyes, and forearm and/or hand. During and after the COVID-19 pandemic, scientists should recognize the importance of contactless PI and strive for the advancement of these technologies to build social and institutional infrastructure.

## Supplementary Information

**Additional file 1. **Preferred Reporting Items for Systematic Reviews and Meta-Analyses extension for Scoping Reviews (PRISMA-ScR) Checklist.

## Data Availability

The data used to support the findings of this study are available from the corresponding author upon request.

## Abbreviations

PI: Personal identification
COVID-19: The coronavirus disease 2019
AI: Artificial intelligence
PRISMA-ScR: The preferred reporting items for systematic reviews and meta-analyses extension for scoping reviews
PCC: Participant-Concept-Context
WHO: The World Health Organization
CNN: Convolutional neural network
